# Conditional knockout of ASK1 in microglia/macrophages attenuates epileptic seizures and long-term neurobehavioural comorbidities by modulating the inflammatory responses of microglia/macrophages

**DOI:** 10.1186/s12974-022-02560-5

**Published:** 2022-08-08

**Authors:** Yiying Zhang, Zhangyang Wang, Rongrong Wang, Lu Xia, Yiying Cai, Fangchao Tong, Yanqin Gao, Jing Ding, Xin Wang

**Affiliations:** 1 Department of Neurology, Zhongshan Hospital, Fudan University, 180 Fenglin Road, Shanghai, 200032 China; 2 Department of the State Key Laboratory of Medical Neurobiology and MOE Frontiers Center for Brain Science, Institutes of Brain Science, Fudan University, Shanghai, China; 3 CAS Center for Excellence in Brain Science and Intelligence Technology, Shanghai, China

**Keywords:** ASK1, Epilepsy, Neurobehaviors, Neuroinflammation, Microglial/macrophage heterogeneity

## Abstract

**Background:**

Apoptosis signal-regulating kinase 1 (ASK1) not only causes neuronal programmed cell death via the mitochondrial pathway but also is an essential component of the signalling cascade during microglial activation. We hypothesize that ASK1 selective deletion modulates inflammatory responses in microglia/macrophages(Mi/Mϕ) and attenuates seizure severity and long-term cognitive impairments in an epileptic mouse model.

**Methods:**

Mi/Mϕ-specific ASK1 conditional knockout (ASK1 cKO) mice were obtained for experiments by mating ASK1^flox/flox^ mice with CX3CR1^creER^ mice with tamoxifen induction. Epileptic seizures were induced by intrahippocampal injection of kainic acid (KA). ASK1 expression and distribution were detected by western blotting and immunofluorescence staining. Seizures were monitored for 24 h per day with video recordings. Cognition, social and stress related activities were assessed with the Y maze test and the three-chamber social novelty preference test. The heterogeneous Mi/Mϕ status and inflammatory profiles were assessed with immunofluorescence staining and real-time polymerase chain reaction (q-PCR). Immunofluorescence staining was used to detect the proportion of Mi/Mϕ in contact with apoptotic neurons, as well as neuronal damage.

**Results:**

ASK1 was highly expressed in Mi/Mϕ during the acute phase of epilepsy. Conditional knockout of ASK1 in Mi/Mϕ markedly reduced the frequency of seizures in the acute phase and the frequency of spontaneous recurrent seizures (SRSs) in the chronic phase. In addition, ASK1 conditional knockout mice displayed long-term neurobehavioral improvements during the Y maze test and the three-chamber social novelty preference test. ASK1 selective knockout mitigated neuroinflammation, as evidenced by lower levels of Iba1^+^/CD16^+^ proinflammatory Mi/Mϕ. Conditional knockout of ASK1 increased Mi/Mϕ proportion in contact with apoptotic neurons. Neuronal loss was partially restored by ASK1 selective knockout.

**Conclusion:**

Conditional knockout of ASK1 in Mi/Mϕ reduced seizure severity, neurobehavioral impairments, and histological damage, at least via inhibiting proinflammatory microglia/macrophages responses. ASK1 in microglia/macrophages is a potential therapeutic target for inflammatory responses in epilepsy.

**Supplementary Information:**

The online version contains supplementary material available at 10.1186/s12974-022-02560-5.

## Introduction

Epilepsy is one of the most common chronic neurological disorders, affecting more than 70 million people worldwide [1–3]. It is characterised by recurrent seizures and a wide range of cognitive and psychosocial impairments [4–6]. Multifactorial mediators, including chronicity, epileptiform discharges, underlying brain disorders and medication, cause cognitive comorbidities in epilepsy [7]. Although several antiseizure medications are in clinical use, 40% of patients still suffer from seizures and cognitive disturbances [8, 9]. In this group of patients, most drugs are ineffective [8, 9], which indicates the need for new therapeutic approaches for epilepsy [10].

Previous pathological studies on epilepsy have mainly focused on neuronal dysfunction, and most antiseizure medications act by modulating ion channels or neurotransmitter systems [11]. Increasing evidence indicates that neuroinflammation is involved in the pathological processes of epilepsy [12–14]. As resident immune cells in the brain, microglia mediate proinflammatory responses. When the brain is injured, bone marrow-derived macrophages invade the brain [15]. Microglia and macrophages become activated in response to epilepsy and contribute to immune responses [16].

Apoptosis signal-regulating kinase 1 (ASK1) has been identified as a mitogen-activated protein kinase kinase kinase (MAP3K) that participates in inflammation, apoptosis and cell proliferation [17, 18]. Previous studies have suggested that ASK1 is highly expressed in patients with epilepsy and in epileptic rat models [19–21]. For instance, Yamamoto et al. [20] detected high levels of ASK1 expression in the hippocampi of patients with temporal lobe epilepsy. Similarly, ASK1 levels increased in the hippocampi of experimental epileptic rats [21]. However, these studies mainly focused on the neuron-specific expression of ASK1 and its role in neuronal apoptosis. Previous studies have rarely explored the relationship between ASK1 and proinflammatory responses initiated and mediated by microglia/macrophages (Mi/Mϕ). In addition, whether ASK1 deletion in Mi/Mϕ mitigated seizure severity or neurobehavioral impairments was not investigated in these studies.

In this work, we investigated the specific temporal and spatial distributions of ASK1 in epileptic mice induced by kainic acid (KA). We explored whether ASK1 selective knockout in Mi/Mϕ alleviated seizure severity and emotional, cognitive impairments by mitigating neuroinflammation.

## Materials and methods

### Animals

The ASK1 conditional knockout mice were ASK1^flox/flox^ (Shanghai Southern Model Biological Corporation Construct). Target transgenic mice (ASK1 cKO) were obtained for experiments after mating ASK1^flox/flox^ mice with CX3CR1^creER^ mice (Jackson Laboratory, stock: 021160) with tamoxifen induction. The mice (8–10 weeks old) were housed in transparent cages at 22–25 °C under a 12 h day-night cycle. The mice had free access to food and water. All experiments were approved by the Ethics Committee of the Fudan University Zhongshan Hospital (Approval number 2019-151, Shanghai, China). The experiments were performed in accordance with the National Institutes of Health Guide for the Care and Use of Laboratory Animals. All animal experiments were reported in compliance with ARRIVE guidelines.

### Establishment of a kainic acid (KA) induced epileptic mouse model

Male mice were randomly divided into four groups: the ASK1 cKO post-kainic acid (cKO KA) group, the wild-type post-kainic acid (WT KA) group, the ASK1 cKO vehicle (cKO sham) group and the wild-type vehicle (WT sham) group. Then, in the post-KA groups, 1.5 µl of KA (0.1 mg/ml, Sigma, USA) was injected into the right dorsal hippocampal region at the following coordinates: anteroposterior (AP), − 1.6 mm; mediolateral (ML), − 1.5 mm; and dorsoventral (DV), − 1.5 mm. In the control groups, 1.5 µl of saline was injected into the hippocampus at the same coordinates. Seizures were monitored for 24 h after KA injection during the acute phase and classified according to the Racine scale[22]. Only mice with stage 3–5 status epilepticus (SE) were included in the following behavioural analysis; other mice were excluded from experiments. After SE, mice exhibited a latent seizure-free phase. Spontaneous recurrent seizures (SRSs) occurred after this latency period[23]. SRSs were monitored with a video surveillance system (Xiaomi, China). The monitoring period began on the 25th day after KA injection and ended on the 32nd day. Only stage 3–5 SRSs were included in our study.

### Pentylenetetrazol (PTZ) induced acute seizures

Pentylenetetrazol (PTZ) (Sigma, USA) was dissolved in normal saline solution at a concentration of 5 mg/ml. PTZ was intraperitoneally injected at a dose of 60 mg/kg. After PTZ administration, all male mice (8–10 weeks old) were monitored for 30 min for acute seizure scoring under video surveillance. The experimenters were blinded to grouping when analyzing the latency to first myoclonic jerk and first generalized tonic–clonic seizure (GTCS) according to the Racine scale.

### Neurobehavioural assessments

#### Three-chamber sociability and social novelty test

As the same of previous studies [24, 25], three-chamber sociability and social novelty preference test was performed in a rectangular box (40 cm × 60 cm × 40 cm) divided into three equal chambers. Empty cages were placed in the left and right chambers to house unfamiliar mice. During the habituation period, a test mouse was placed in the middle chamber and allowed to freely explore three chambers for 5 min. Following acclimation, a strange mouse (stranger 1, gender and age-matched) was placed in the cage in the left or right chamber, and the experimental mouse was allowed to freely explore all three chambers for 15 min. In this three-chamber sociability test, the amount of time spent near stranger 1 and the empty cage was recorded. If the test mouse spent more time exploring the empty cage than stranger 1, this mouse might have impaired sociability, preventing it from communicating with unfamiliar mice.

In the three-chamber social novelty preference test, another strange mouse (stranger 2, gender and age-matched) was placed in the cage in the opposite compartment, and the test mouse was allowed another 15 min to explore the already-investigated, now-familiar mouse (stranger 1 from the sociability test) and unfamiliar stranger 2. The amount of time spent near stranger 1 and stranger 2 was recorded. If the experimental mouse spent more time exploring stranger 1 than stranger 2, the mouse might have impaired social novelty recognition.

#### Open field test

The open field test was performed in an arena (40 × 40 × 40 cm), with test mouse placed in the centre and allowed to explore the arena for 10 min. The average speed and the amount of time spent in the central area were recorded. The percentage of time spent in the central area was compared among the four groups. Lower central time indicated anxiety and depression-like behaviours in experimental mice.

#### Y maze

All mouse groups were subjected to the Y maze test to assess working memory impairment. The maze had three arms, each measuring 30 × 6 × 15 cm. During the test, a test mouse was placed in one arm and allowed to freely move from one arm to the others for 10 min. The alternation rate was calculated by software as follows: actual alternation/maximum alternation*100%. The actual alternation value was the number of times that the mouse went into the three arms in a row. The maximum alternation value was the total number of arm entries minus 2. In the Y maze test, a lower alternation rate suggested impaired working memory.

#### Morris water maze test

The Morris water maze was conducted in a circular pool (110 cm in diameter) filled with water at 20–22 °C, with a platform (10 cm in diameter) submerged 1 cm below the water. This test included 3 days of spatial acquisition training before KA injection, followed by 5 days of similar training and a final probe test for mice with chronic KA-induced epilepsy.

During the spatial acquisition trials (5 consecutive days), each mouse was placed in four different starting positions each day and given 60 s to find the platform. If the mouse failed to reach the platform in this time, the mice were guided to the platform. After the mouse found the platform, it was held on the platform for 15 s. The escape latency was recorded by software. A longer escape latency suggested a weaker learning ability.

During the probe test, the platform was removed, and the mouse was allowed to swim in the pool for 60 s. The number of times that the mouse crossed the platform and the amount of time spent in the target quadrant were calculated. If the test mouse had an impaired memory, it spent little time in the target quadrant and did not pass over the platform often.

### Quantitative reverse transcriptase PCR

Total RNA from the hippocampus was extracted using a Yishan kit (Yishan, Shanghai, China). Complementary DNA was generated using a thermo kit (Thermo, United States)). Quantitative PCR was conducted using SYBR (Yeason, Shanghai, China) on a Thermal Cycler Dice.

The primers for RT-qPCR were shown as followed:

CD16 F: TTTGGACACCCAGATGTTTCAG, R: GTCTTCCTTGAGCACCTGGATC

CD32 F: AATCCTGCCGTTCCTACTGATC, R: GTGTCACCGTGTCTTCCTTGAG

TNF-α F: GACCCTCACACTCAGATCATCTTCT,R: CCTCCACTTGGTGGTTTGCT

IL-1β F: CTCCATGAGCTTTGTACAAGG, R: TGCTGATGTACCAGTTGGGG

IL-6 F: ACACATGTTCTCTGGGAAATC, R: AGTGCATCATCGTTGTTCATA

Arg1 F: TCACCTGAGCTTTGATGTCG, R: CTGAAAGGAGCCCTGTCTTG

CCL22 F: CTGATGCAGGTCCCTATGGT, R: GCAGGATTTTGAGGTCCAGA

IL13 F: CCTGGCTCTTGCTTGCCTT, R: GGTCTTGTGTGATGTTGCTCA

TGFβ F: TGCGCTTGCAGAGATTAAAA, R: CGTCAAAAGACAGCCACTCA

Ym1/2 F: CAGGGTAATGAGTGGGTTGG, R: CACGGCACCTCCTAAATTGT

CD68 F:GAAATGTCACAGTTCACACCAG, R:GGATCTTGGACTAGTAGCAGTG

C3 F: CAAAGGACCTGGAACTGCTGG, R: GGTCAGGCAGTCTTCTTCGG

GAPDH F: GTGAAGGTCGGTGTGAACGG,R: GTTTCCCGTTGATGACCAG (GAPDH was used as an internal control)

### Western blot analysis

Total protein from the hippocampus was extracted with commercial extraction kits (Thermo Fisher Scientific Corporation, United States). Protein lysates were separated by SDS-PAGE gels and transferred to polyvinylidene fluoride (PVDF) membranes (Millipore Corporation, United States).

The membranes were blocked with 5% bovine serum albumin (BSA) for 90 min at 37 °C. Then, the membranes were incubated overnight at 4 °C with the following primary antibodies: rabbit anti-pASK1 (1:500, Cell Signaling Technology, MA, United States), rabbit anti-ASK1 (1:1000, Abcam, United States), and mouse anti-β-actin (1:2000, Proteintech, Wuhan, China). The next day, the membranes were incubated with horseradish peroxidase-conjugated goat anti-rabbit or anti-mouse secondary antibodies (1:2000, Proteintech, Wuhan, China). Protein bands were visualized using an enhanced chemiluminescence (ECL) reagent (Yeason, Shanghai, China) and a Bio–Rad image analysis system.

### Immunofluorescence staining

Brain tissues were fixed with 4% paraformaldehyde for 24 h and then incubated with 20% and 30% sucrose solutions for 24 h. Next, the brains were sectioned into 25 μm sections, and the sections were washed 3 times with a phosphate-buffered saline (PBS) solution. After washing, the sections were permeabilized with 1% Triton X-100 and blocked with donkey serum for 90 min, followed by incubation with primary antibodies overnight at 4 °C. The primary antibodies used included: rabbit anti-ASK1 (1:100, Abcam, US), goat anti-Iba1 (1:1000, Novus, US), mouse anti-NeuN (1:2000, Abcam, US), rat anti-CD16 (1:100, BD, US), rabbit anti-CD206 (1:200, Abcam, US), rabbit anti-pJNK (1:200, Affinity, China), rabbit anti-pp38 (1:200, Affinity, China), rabbit anti p–c jun(1:200, Cell Signaling Technology, US) and rat anti-CD68(1:500, Bio-Rad, US). The next day, the sections were incubated with secondary antibodies in the dark for 60 min at 37 °C. The following secondary antibodies were used: Alexa Fluor 594-labelled goat anti-rabbit IgG (Proteintech; 1:1000), fluorescein isothiocyanate (FITC)-conjugated goat anti-mouse IgG (Proteintech; 1:1000), and Alexa Fluor 647-labelled goat anti-mouse IgG (Proteintech; 1:1000). Finally, the immunofluorescently labelled sections were captured using a laser scanning confocal microscope (Nikon, Germany).

### TUNEL staining

Terminal deoxynucleotidyl transferase dUTP nick end labelling (TUNEL) staining(Roche, US) was used to assess DNA damage. Slices were rinsed twice with PBS and permeabilized with 1% Triton X-100 for 10 min at 4 °C. Then, slices were washed with PBS. After incubation with TUNEL reaction mixture for 60 min at 37 °C in the dark, slides were washed with PBS. Finally, the TUNEL stained sections were captured using confocal microscope (Nikon, Germany).

### Skeleton analysis

Z-stack confocal images (25 μm) were acquired at 0.5um intervals using a confocal microscope (Nikon, Germany). The Image Fiji software was used to convert Z-stack images into a maximum intensity projection image, to convert the image to binary and skeletonize the image. The detailed steps were depicted by Young K et al. [26]. After running the plugin Analyze Skeleton(2D/3D), data from each image (total length of microglial processes and total number of microglial process endpoints) were collected. Some data (2 endpoints with a maximum branch length less than the cutoff value) should be trimmed to remove skeleton fragments.

### Three-dimensional reconstruction

The 25-μm coronal slices were imaged on a confocal microscope (Nikon, Germany) using an oil objective. Imaging parameters were consistent across all experiments. We performed Z stacking with 1.0-μm steps in the z direction. The three-dimensional reconstruction of the representative images was performed with Imaris 9.0.1 software (Bitplane).

### Statistical analysis

GraphPad Prism software was used to conduct the statistical analysis. The Shapiro–Wilk test was used to determine the normality of the data distribution. Comparisons between the two groups were performed using the Student’s t test (normal distributions) or the Mann–Whitney *U* test (non-normal distributions). Multiple group analyses were performed by one- or two-way ANOVA followed by a Tukey post hoc test for normal distributions, while for nonnormal distributions, comparisons were performed using Kruskal–Wallis ANOVA followed by Dunn’s post hoc test. Experimental data are presented as the mean ± standard deviation (SD). *p* < 0.05 was considered statistically significant.

## Results

### The expression of ASK1 in epileptic mice

**Fig. 1:**
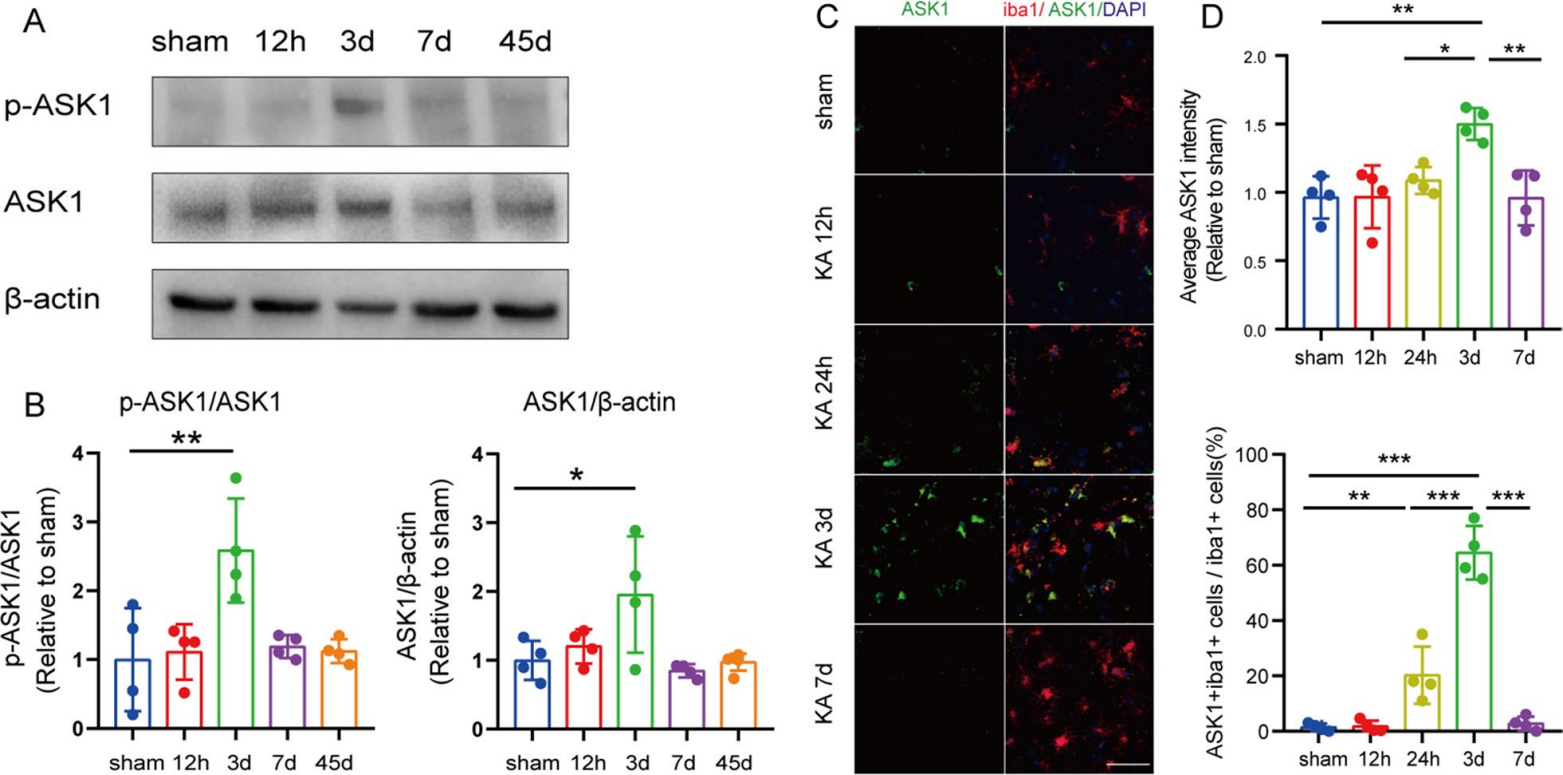
The expression of ASK1 from 12 h to 45 d in epileptic mice. **A** Representative bands of the Western blot in the ipsilateral hippocampus. **B** Quantitation of phosphate-ASK1 and total ASK1 expression of ipsilateral hippocampus in Western blot. **C** Representative images of ASK1 (green) in Mi/Mϕ (Iba1, red) immunofluorescence staining following different timepoints (12 h, 1d, 3d, 7d). Scale bar = 50 µm. **D** Quantitation of ASK1 intensity of staining and the percentage of ASK1^+^Iba1^+^ cells. All data are expressed as the mean ± SD and analyzed using the one way ANOVA. ***p < 0.001, **p < 0.01, and *p < 0.05, *vs.* sham group. *n* = 4/group

Several studies have reported that ASK1 levels were increased in patients with epilepsy and in epileptic rats [21]. To explore ASK1 levels induced by KA in our epileptic mice, western blotting was performed. Significantly increased p-ASK1 and ASK1 levels were detected in the hippocampus of epileptic mice on the third day after KA injection (*n* = 4 per group; *p* < 0.05) (Fig. 1A, B). We conducted double immunofluorescence staining of microglia/macrophage marker Iba1 and ASK1 in the hippocampus of both epileptic and sham mice in the following timepoints: 12 h, 1d, 3d, 7d (Fig. 1C). Our results indicated that the expression of ASK1 significantly increased on day 3, but not day 1, after KA injection comparing with the sham group (Fig. 1D). And the increased ASK1 almost was localized in the Mi/Mϕ in the hippocampus 3 days after intrahippocampal KA injection (Fig. 1D). This indicated that ASK1 was highly expressed in Mi/Mϕ in the hippocampus 3 days post seizures. ASK1 was also colocalized with Mi/Mϕ in the cortex and striatum on day 3 (Additional file 1: Fig. S1).

### ASK1 conditional knockout alleviated the severity of acute and chronic seizures in mice

**Fig. 2:**
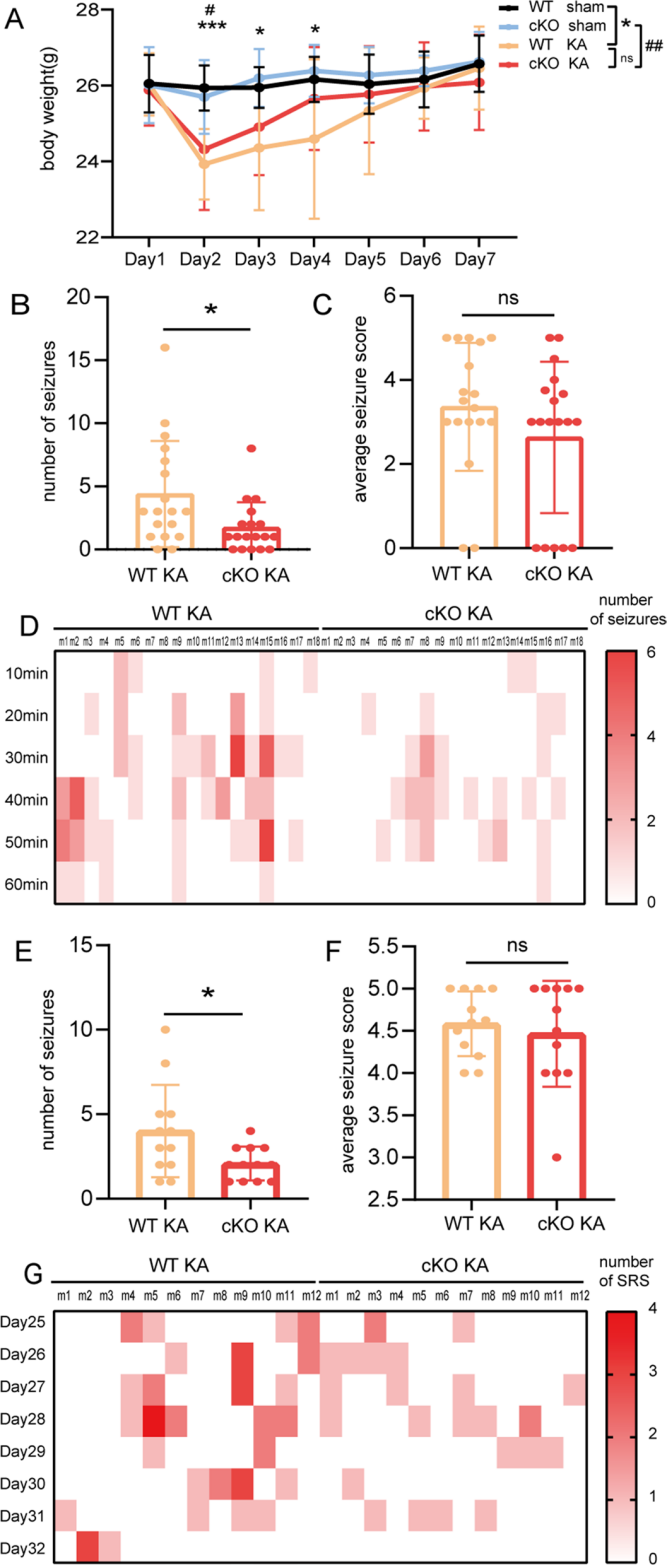
The effect of ASK1 cKO on acute and chronic seizures after KA injection. **A** ASK1 knockout in mice did not affect weight. **B**, **C** ASK1 cKO downregulated the frequency of acute seizures (**B**), but did not affect the average seizure score **C** in the acute period. **D** The number of seizures in the acute period in the WT KA and cKO KA groups is displayed in the form of a heatmap, with a darker color indicating more seizures. **E**–**F** ASK1- cKO reduced the total number of SRSs (**E**), but the average seizure score **F** was not affected by ASK1 cKO. **G** The number of SRSs in the WT KA and cKO KA groups for 8 consecutive days (Day 25 to Day 32 after KA injection) is displayed in the form of a heatmap, with a darker color indicating more SRSs. All data are expressed as the mean ± SD. ***p < 0.001, *p < 0.05, WT KA group *vs.* WT Sham group, or as indicated; ^##^*p* < 0.01, and ^#^*p* < 0.05, cKO KA group *vs.* cKO sham group. *n* = 8 in the WT sham or cKO sham groups, *n* = 12 in WT KA or cKO KA groups, *p* values are based on the two way ANOVA test (**A**). *n* = 18 in WT KA and cKO KA groups in the acute period (**B**–**D**), and *n* = 12 in WT KA and cKO KA group in the chronic period (**E**–**G), p** values are based on the Student’s *t* test or Mann–Whitney *U* test (**B**–**F**)

Previous reports have suggested that ASK1 inhibitors could play a protective role against stroke, which led us to assess the effect of ASK1 genetic deletion on epilepsy [27, 28]. To explore the efficiency of ASK1 cKO, we sorted microglia/macrophages and extracted mRNA for PCR and checked it by agarose gel electrophoresis. Our results suggested that ASK1 was not detected in agarose gel electrophoresis after ASK1 cKO. We also conducted double immunofluorescence staining of Iba1/ASK1 3 days after KA injection, our results showed that ASK1 cKO significantly reduced ASK1 expression in Mi/Mϕ (Additional file 1: Fig. S2). The weight of mice after KA injection was recorded for 7 consecutive days. No notable differences were observed between the WT KA and cKO KA groups, which indicated that ASK1 conditional knockout had no effect on body weight (Fig. 2A). To explore the effect of ASK1 cKO on acute seizure severity, we used behaviour video recordings after KA injection for seizure measurements. The mice recovered immediately after being removed from isoflurane anaesthesia and gradually developed seizures. ASK1 genetic deletion in Mi/Mϕ significantly decreased acute seizure frequency (*p* = 0.0211) (Fig. 2B, D) but did not affect seizure scores in epileptic mice (*p* = 0.2015) (Fig. 2C). During the chronic phases, ASK1 deficiency clearly reduced the frequency of spontaneous recurrent seizures (SRSs) (*p* = 0.0324) (Fig. 2E, G), but seizure scores between Days 25–32 remained unchanged (*p* = 0.5815) (Fig. 2F). These results demonstrated that ASK1 deletion alleviated the frequency of KA-induced acute and chronic seizures. To further explore whether ASK cKO showed similar anti-seizure effects in other seizure models, such as PTZ induced acute seizures (60 mg/kg, ip) in male mice, experimenters recorded the latency to first myoclonic jerk and first GTCS. These experimenters were blinded to grouping. The results indicated that ASK1 cKO significantly increased the latency of PTZ induced seizures (Additional file 1: Fig. S3).

### ASK1 conditional knockout alleviated impaired social novelty recognition and spatial working memory in epileptic mice

Emotional and cognitive difficulties are common in patients with epilepsy [29–32]. To investigate these neurobehavioural comorbidities in an animal model, epileptic mice were subjected to three-chamber sociability and social novelty preference tests, the open field test, the Y-maze test and the water maze test between Days 33–44 after KA administration.

#### ASK1 conditional knockout reduced abnormal social and stress related activity in epileptic mice

**Fig. 3:**
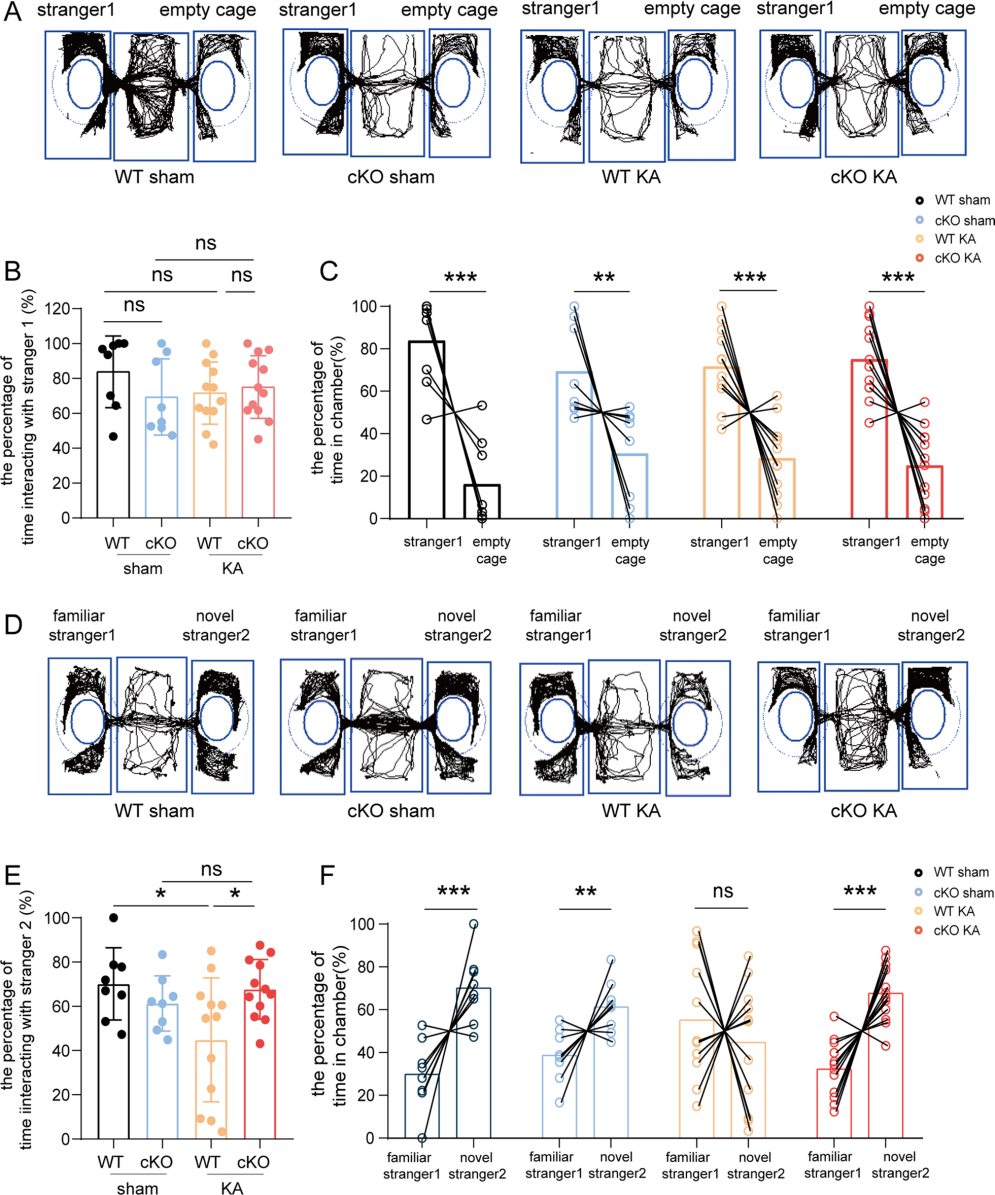
The effect of ASK1 cKO on social and stress related activity after KA injection. The three-chamber sociability and social novelty preference test was used to determine whether epileptic mice had normal sociability **A**–**C** and normal social novelty recognition(D-F). **A** Representative track chart of the three-chamber sociability test was displayed. **B** Quantification of the interacting time with stranger 1. **C** Quantification of the proportion of time spent with stranger 1 or near empty cage. **D** Representative track chart of the three-chamber social novelty preference test. **E** Quantification of the time interacting with the stranger 2. **F** Quantification of proportion of time interacting with stranger 1 or stranger 2. All data are expressed as the mean ± SD. ***p < 0.001, **p < 0.01, and *p < 0.05, *ns* no significance, as indicated. *n* = 8 in the WT or cKO sham groups, *n* = 12 in WT KA or cKO KA groups, *p* values are based on the one way ANOVA test (**B**, **E**), Student’s *t* test (**C**, **F**)

The three-chamber sociability and social novelty preference test was performed to determine whether epileptic mice had abnormal social and stress related activity. In this test, empty cages were placed in the left and right chambers to house unfamiliar mice. During the habituation period, a test mouse was placed in the middle chamber and allowed to freely explore for 5 min. During the three-chamber sociability test, a strange mouse (stranger 1, gender and age-matched) was placed in the cage in the left or right chamber, and the test mouse was allowed to freely explore all three chambers for 15 min. Both WT and cKO epileptic mice frequently interacted with stranger 1 rather than the empty cage, which indicated that WT and cKO epileptic mice had normal sociability (Fig. 3A–C). While in the three-chamber social novelty preference test, WT epileptic mice avoided communicating with a novel stranger (stranger 2). The preference for an already-investigated, now-familiar mouse (stranger 1 from the sociability test) over stranger 2 in WT epileptic mice indicated impaired social novelty recognition in the WT KA group. However, in the cKO KA group, epileptic mice showed normal social novelty recognition (that is, more interactions with stranger 2 than with stranger 1). The cKO KA group had significantly more contact with stranger 2 when compared to WT KA group (*p* = 0.0326) (Fig. 3D–F). Our observations suggested that ASK1 deletion reduced abnormal social and stress related activity in epileptic mice.

#### ASK1 conditional knockout alleviated impaired spatial memory in epileptic mice

**Fig. 4:**
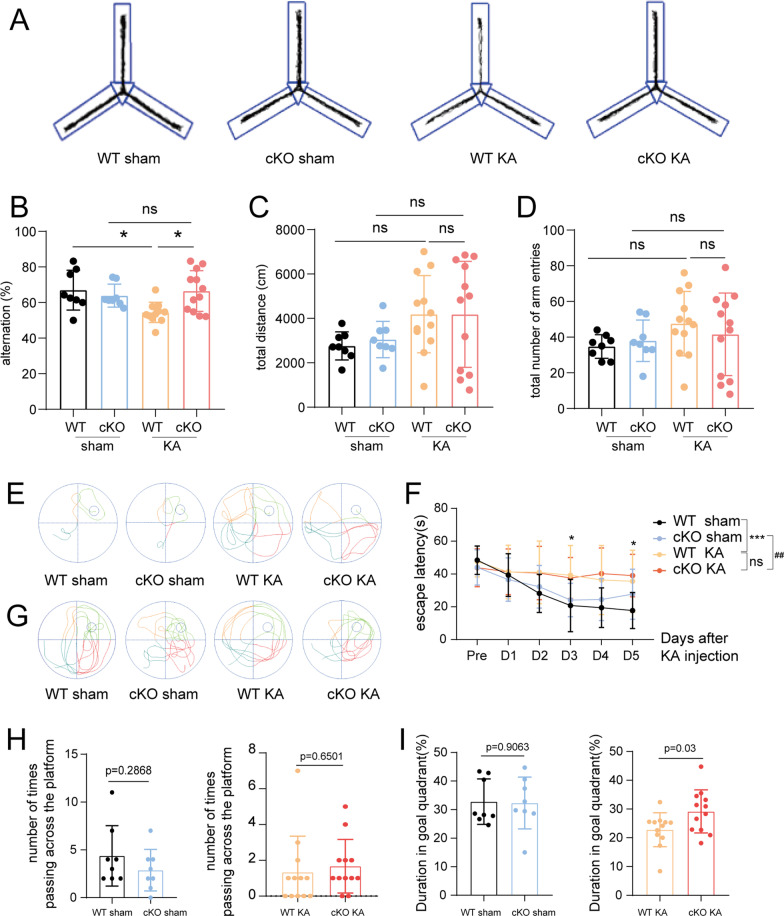
The effect of ASK1 cKO on cognitive functions after KA injection. **A** The Y maze test was performed to detect working memory function. Representative track chart of Y maze test. **B**–**D** Quantification of the alternation rate (**B**), the total travelled distance (**C**), and the total number of arm entries (**D**). **E**, **F** The water maze test was conducted to explore the learning ability. Representative swim paths (**E**), and latency to find the hidden platform (**F**). **G**–**I** The water maze test was conducted to explore the cognition. Representative track chart of the probe test was displayed (**G**), quantification of the numbers of platform crossings **H** and time spent in the goal quadrant (**I**). All data are expressed as the mean ± SD. ***p < 0.001, *p < 0.05, ^##^*p* < 0.01, *ns* no significance, as indicated. *n* = 8 in the WT sham or cKO sham groups, n = 12 in the WT KA or cKO KA groups. *p* values are based on the one way ANOVA (**B**–**D**), two way ANOVA (**F**), Student’s *t* test (**H**, **I**)

Impaired spatial working memory was assessed with the Y maze test. During this test, an experimental mouse was allowed to freely move from start arm to the other two arms for 10 min. The percentage of spontaneous alternation was calculated as follows: actual alternation/maximum alternation*100%. The actual alternation value was the number of times that the mouse went into the three arms in a row. In the Y maze test, normal mice remembered the previously visited maze arms and preferred to explore previously unvisited arm rather than familiar one, however, experimental mice with impaired working memory might forget already-visited arms and enter the recently visited one. Under these circumstances, a lower alternation rate in the Y maze test suggested impaired working memory. In our study, epileptic mice had lower spontaneous alternation rate compared to sham mice (KA vs. control, *p* = 0.0231). Interestingly, ASK1 conditional deletion increased the percentage of spontaneous alternation (cKO KA vs. WT KA, *p* = 0.0133) (Fig. 4A, B). In addition, the total distance travelled and the total number of arm entries were comparable across the four groups, indicating that all four groups had similar motor abilities (Fig. 4C, D). Based on the above results, we assumed that epileptic mice had impaired spatial working memory, and that ASK1 deletion partially improved this memory impairment.

The water maze test was performed to assess learning ability and spatial memory in epileptic mice. This test included spatial acquisition trials (5 consecutive days) as well as a final probe test. During the spatial acquisition trainings, each mouse was placed in four different starting positions and given time to find the platform. The average time to find the platform was called escape latency. A longer escape latency suggested a weaker learning ability. In our study, WT epileptic mice spent more time finding the platform than the controls, and, compared with the WT KA group, ASK1 deletion did not reduce the escape latency time, which suggested that both WT and cKO epileptic mice have impaired learning abilities (Fig. 4E, F). During the probe test (day 6), the platform was removed from the target quadrant, and the experimental mouse was allowed to swim in the pool for 60 s. Representative track chart of the probe test was shown in Fig. 4G. The number of times crossing the platform region and the amount of time spent in the target quadrant were calculated. Both WT KA and cKO KA groups failed to cross the platform region as frequently as the control groups (Fig. 4H). However, the percentage of time swimming in the target quadrant was higher in the cKO KA group than that in the WT KA group (cKO KA vs. WT KA, *p* = 0.03) (Fig. 4I). This might indicate less impaired memory in the cKO KA group than in the WT KA group. Although cKO epileptic mice did not cross platform region more often than WT epileptic mice, these cKO epileptic mice spent more time swimming in target quadrant to search for the already-removed platform.

Both results of Y maze test and water maze test reflected that ASK1 conditional knockout alleviated impaired spatial memory in epileptic mice.

#### ASK1 conditional knockout failed to reduce anxiety and depression-like behaviours in epileptic mice

Anxiety and depression-like behaviours were recorded in the open field test. In this test, the experimental mouse was allowed to explore the arena for 10 min. Normal mice freely explored the central and surrounding area, while WT epileptic mice were reluctant to explore the central area, and spent less time in the central area of the arena than control mice (*p* = 0.0192). ASK1 conditional deletion did not increase the central time (cKO KA vs. WT KA, *p* = 0.9591) (Additional file 1: Fig. S4A, B). Less time in the central area was associated with higher levels of anxiety and depression, these results suggested that ASK1 deficiency failed to reduce anxiety and depression-like behaviours in epileptic mice. In addition, the average speed was comparable across all groups, indicating that there were no considerable differences in motor abilities.

Consistent with the literature [33, 34], a variety of cognitive, emotional, and social related behaviors of rodents may not be displayed sensitively. Therefore, we used a variety of behavioral tests to assess the effect of ASK1 cKO on the behavioral phenotypes. We found that ASK1 KO in Mi/Mϕ significantly reduced abnormal social and stress related activity and alleviated impaired spatial memory in epileptic mice, but failed to reduce anxiety and depression-like behaviors in epileptic mice. Our results suggested that ASK1 cKO improved some neurobehavioral outcomes, especially in the three-chamber social novelty preference test and Y maze test.

### ASK1 conditional knockout altered the polarization of Mi/Mϕ towards pro- or anti-inflammatory phenotypes on day3 post seizures

**Fig. 5:**
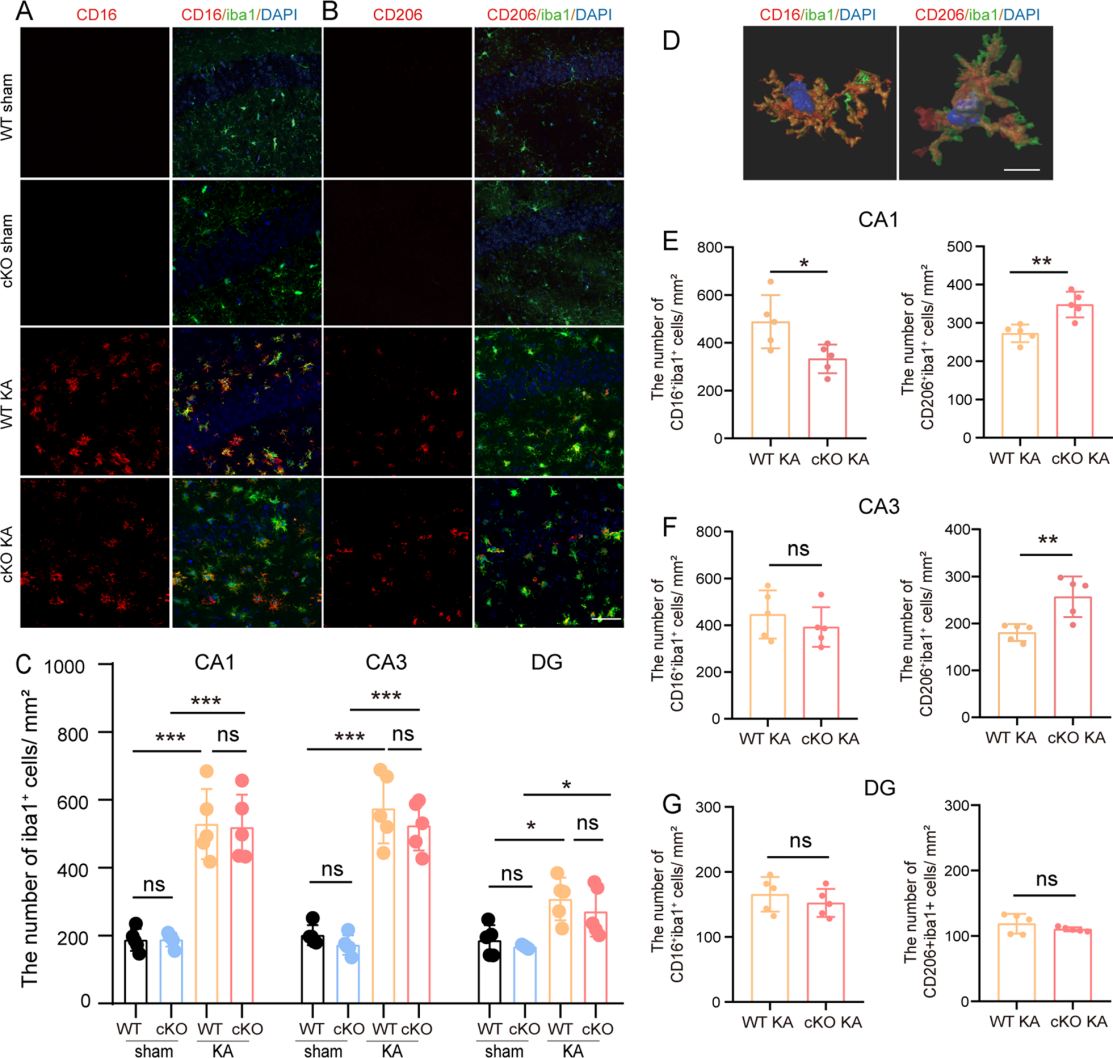
ASK1 cKO affected polarization of Mi/Mϕ in the hippocampus on day3 after KA administration. **A**, **B** Representative images of pro-inflammatory Mi/Mϕ (CD16/Iba1, red/green) **A** or anti-inflammatory Mi/Mϕ (CD206/Iba1, red/green) **B** immunostaining in the CA1, CA3, and DG regions of the hippocampus. Scale bar = 50 µm. **C** Quantification of the total number of Iba1^+^ cells. **D** Three-dimensional reconstruction of representative pro- and anti-inflammatory Mi/Mϕ was performed with Imaris 9.0.1 software. Scale bar = 10 µm. **E**–**G** Quantification of CD16^+^/Iba1^+^ or CD206^+^/Iba1^+^ expression in the CA1(E), CA3 (**F**), and DG **G** region. All data are expressed as the mean ± SD.***p < 0.001, **p < 0.01, and *p < 0.05, ns:no significance, as indicated. *n* = 5/group. *p* values are based on the one way ANOVA (**C**), Student’s *t* test(**E**–**G**)

Since behavioural improvements were observed after ASK1 conditional deletion, we explored whether there were histological alterations between the WT KA and cKO KA groups. In our study, we skeletonized Mi/Mϕ, and measured the length of processes, the number of process endpoints of Mi/Mϕ. ASK1 conditional deletion reduced the total length of processes(*p* = 0.0462), while did not alter the number of process endpoints when compared to those in WT KA group (Additional file 1: Fig. S5). We conducted immunofluorescence staining of Mi/Mϕ marker Iba1 to measure the total number of Mi/Mϕ 3 day post seizures (Fig. 5A, B). No remarkable difference was detected in the hippocampus between the WT KA and cKO KA groups (Fig. 5C). We further detected the densities of Iba1^+^/CD16^+^ cells and Iba1^+^/CD206^+^ cells. CD16 was a specific cell marker of pro-inflammatory Mi/Mϕ, while CD206 was a cell marker of anti-inflammatory Mi/Mϕ (Fig. 5D) [35, 36]. ASK1 conditional deletion reduced the density of Iba1^+^/CD16^+^ cells (cKO KA vs. WT KA, *p* = 0.0250) and increased the density of Iba1^+^/CD206^+^ cells (cKO KA vs. WT KA, *p* = 0.0033) in the CA1 region (Fig. 5E). Moreover, in the hippocampal CA3 area, the density of Iba1^+^/CD206^+^ cells was substantially higher in cKO epileptic mice than in WT epileptic mice (cKO KA vs. WT KA, *p* = 0.0067) (Fig. 5F). In the DG region, the densities of Iba1^+^/CD16^+^ cells and Iba1^+^/CD206^+^ cells were similar between cKO and WT epileptic mice (Fig. 5G).

These results suggested that ASK1 deficiency inhibits the pro-inflammatory responses and promotes anti-inflammatory responses of Mi/Mϕ.

### ASK1 conditional knockout altered the mRNA expression of pro- and anti-inflammatory markers on day3 in the hippocampus

**Fig. 6:**
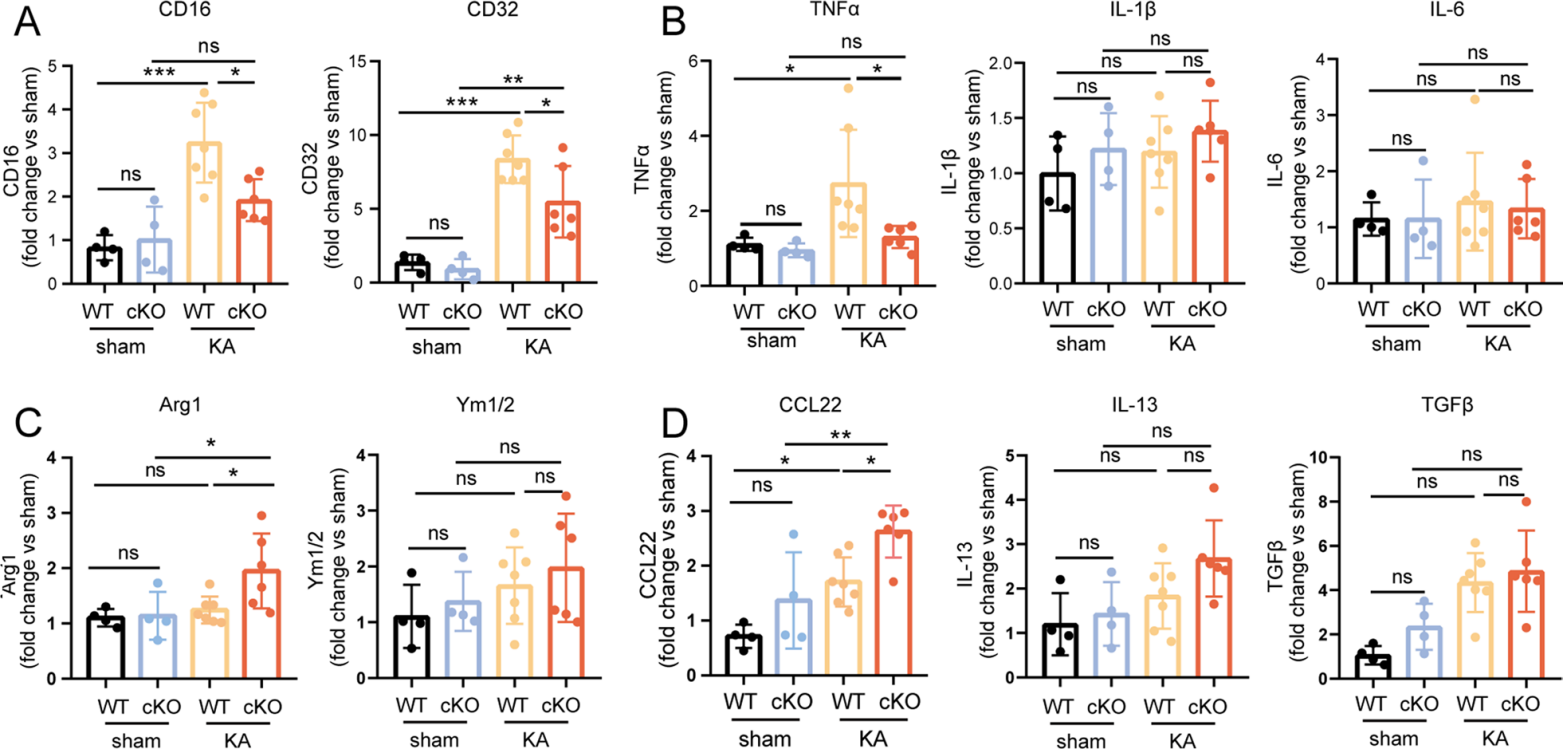
ASK1 cKO altered the mRNA expression of pro- and anti-inflammatory markers in the hippocampus(3d). **A** The mRNA levels of pro-inflammatory Mi/Mϕ specific marker (CD16, CD32). **B** The mRNA levels of released pro-inflammatory cytokines (TNFa, IL-1β and IL-6). **C** The mRNA levels of anti-inflammatory Mi/Mϕ specific markers (Arg-1, Ym1/2). **D** The mRNA levels of released anti-inflammatory cytokines (IL-13, TGFβ) and chemokines (CCL22). All data are expressed as the mean ± SD. ***p < 0.001, **p < 0.01, and *p < 0.05, *ns* no significance, as indicted. *n* = 4 in the WT sham or cKO sham groups, *n* = 7 in the WT KA group, *n* = 6 in the cKO KA group. *p* values are based on the one way ANOVA and Kruskal–Wallis ANOVA(A-D)

To further confirm the inflammation of brain by Mi/Mϕ-induced in an epileptic mouse model, we examined the messenger RNA (mRNA) expression of pro- or anti-inflammatory Mi/Mϕ related markers in the hippocampus with RT–qPCR. Pro-inflammatory Mi/Mϕ expressed specific cell markers (CD16, CD32) (Fig. 6A), and released inflammatory cytokines including tumour necrosis factor α (TNFα), interleukin-1 β (IL-1 β), and interleukin-6(IL-6) (Fig. 6B). While anti-inflammatory Mi/Mϕ expressed cell marker including arginase 1 (Arg1), chitinase-like protein (Ym1/2) (Fig. 6C), and secreted anti-inflammatory cytokines including interleukin-13(IL-13), transforming growth factor-β(TGFβ) and chemokines such as CC chemokine ligand 22 (CCL22) (Fig. 6D) [37, 38]. In our study, KA injection increased the mRNA expression of both pro- and anti-inflammatory markers in the hippocampus comparing with the control groups. ASK1 conditional deletion reduced the expression of CD16 (*p* = 0.0154), CD32 (*p* = 0.0291), and TNFα (*p* = 0.0404) and increased the levels of CCL22 (*p* = 0.0300) and Arg1 (*p* = 0.0456) in the hippocampus (Fig. 6A–D).

### ASK1 cKO increased the Mi/Mϕ distribution close to apoptotic neurons on day3

**Fig. 7:**
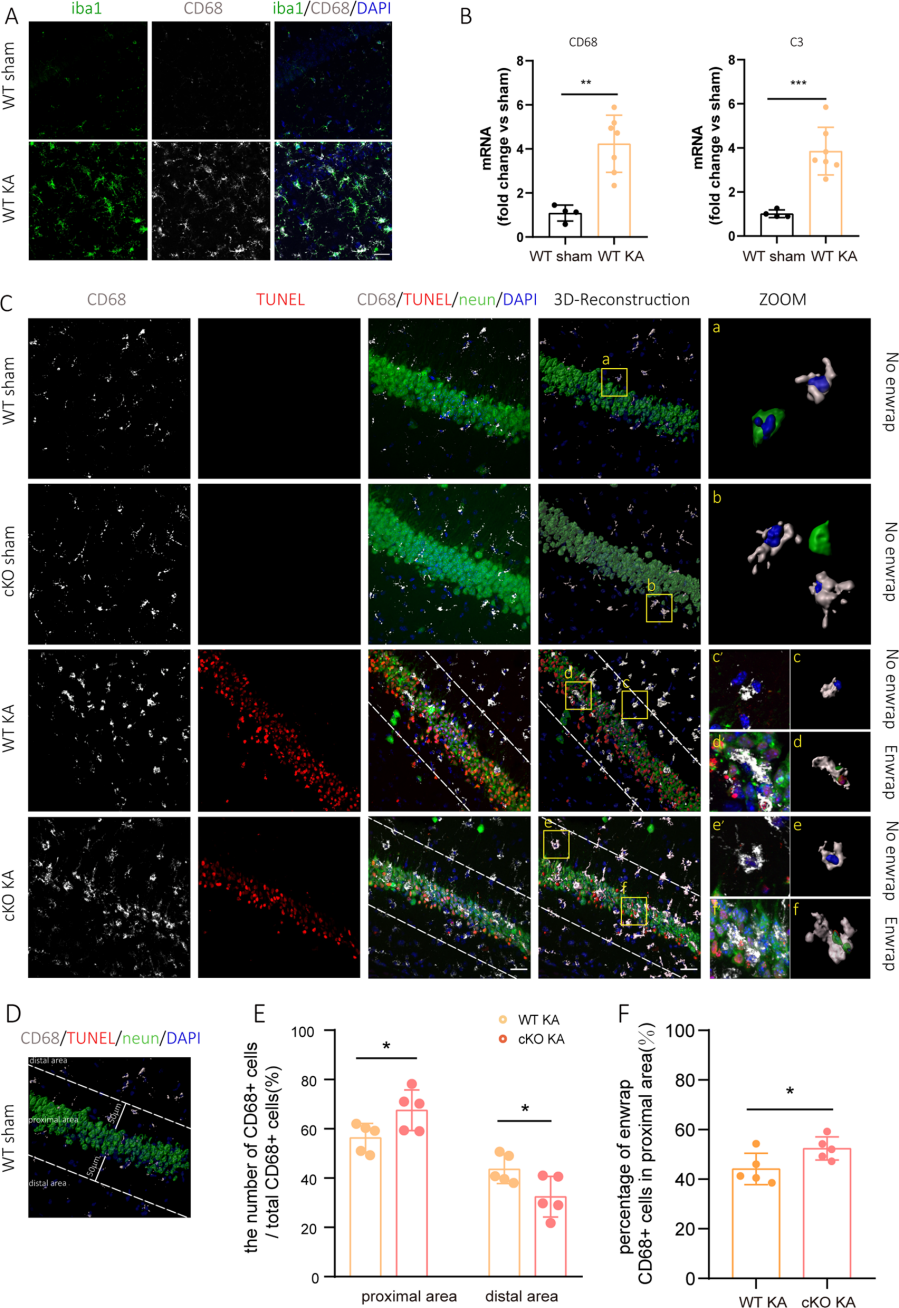
ASK1 cKO increased the Mi/Mϕ distribution close to apoptotic neurons 3 day after KA injection. **A** Representative images of Iba1 (green) and CD68 (grey) immunofluorescence staining in the hippocampus. Scale bar = 30 µm. **B** Quantification of the mRNA levels of C3 and CD68, n = 4 in the WT sham group, *n* = 7 in the WT KA group. **C** Representative images of TUNEL (red) staining, NeuN(green) and CD68 (grey) immunostaining in the hippocampus (blue staining indicates DAPI). Scale bar = 30 µm. Three-dimensional reconstruction of representative CD68^+^/neun^+^ /TUNEL^+^ cells by Imaris 9.0.1 software. **D** Illustrates the proximal area and distal area. **E** Quantification of the Mi/Mϕ density in the proximal, distal area. **F** Quantification of enwrapping (NeuN^+^ TUNEL^+^ CD68^+^) Mi/Mϕ in proximal area. Small plots of the yellow square boxes (a–f) were zoomed to show "Enwrap" state of CD68^+^ cells. All data are expressed as the mean ± SD. ***p < 0.001, **p < 0.01, and *p < 0.05, as indicated. *n* = 5 in each group. *p* values are based on the Student’s *t* test (**B**, **E**, **F**)

Microglial activation occurring early after brain injury is widely believed. Both proinflammatory and anti-inflammatory Mi/Mϕ express phagocytic receptors [39, 40]. They redistribute in the brain, move towards the damaged area, and phagocyte myelin debris, abnormal protein aggregation and apoptotic neurons. Previous studies suggested that anti-inflammatory Mi/Mϕ may display a stronger ability to phagocyte dead neurons when compared to proinflammatory Mi/Mϕ [41, 42]. In our study, we investigated whether ASK1 conditional deletion altered the chemotaxis and phagocytosis functions of Mi/Mϕ. In our experiments, increased colocalization of Iba1 with CD68, a microglial lysosomal marker, indicated increased phagocytotic activity of the reactive Mi/Mϕ after KA injection (Fig. 7A). The complement pathways are responsible for Mi/Mϕ-mediated phagocytosis, increased mRNA levels of C3(*p* = 0.0006) and CD68 (*p* = 0.0012) also suggested increased phagocytosis in KA group when compared to those in sham group (Fig. 7B).

To determine the spatial distribution of Mi/Mϕ relative to apoptotic neurons in the hippocampus in WT KA and cKO KA groups, staining of TUNEL, NeuN and CD68 in the hippocampus were conducted, and the 3D-reconstruction method was used (Fig. 7C). We detected the distribution and phagocytosis activity of microglia/macrophages in the hippocampus. We have drawn white dotted lines 50 µm around the hippocampal neurons’ boundaries (Fig. 7D), the area between the two white dotted lines was defined as proximal area and the other area was defined as the distal area (more than 50 µm away from the hippocampal neurons’ boundaries). ASK1 cKO markedly increased the Mi/Mϕ density in proximal area, and decreased the Mi/Mϕ distribution in distal area comparing with the WT group post KA injection (Fig. 7E). In proximal area, we found some CD68 positive cells enwrapping apoptotic neurons. "Enwrap" mainly refers to the state that CD68 + Mi/Mϕ surround apoptotic neurons. CD68 is a microglial lysosomal marker, and indicates strong phagocytotic activity in Mi/Mϕ [43–45]. We counted the proportion of CD68 positive cells enwrapping apoptotic neurons in proximal area. The percentage of these "Enwrap" state CD68 positive cells significantly increased in proximal area after ASK1 cKO, which might indicate increased phagocytosis of apoptotic neurons in cKO KA group (Fig. 7F).

### ASK1 conditional knock out alleviated loss of neurons

**Fig. 8:**
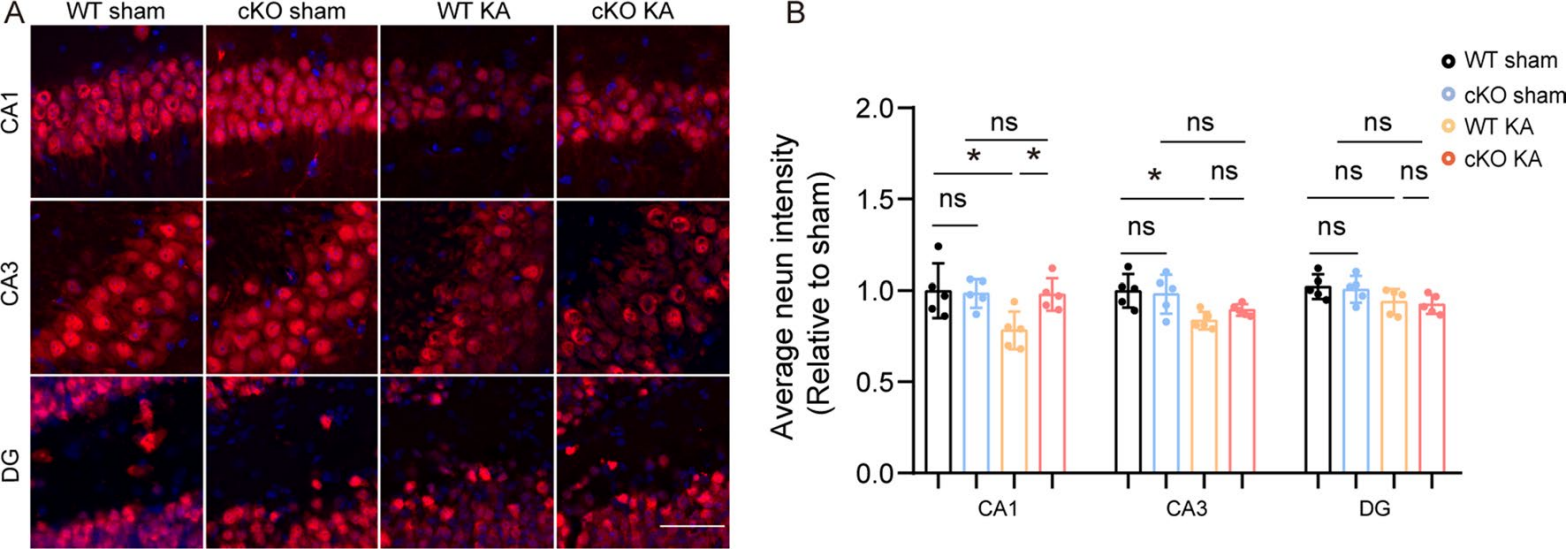
ASK1 cKO partially alleviated neuronal damage on the 7th day after KA injection. **A** Representative images of NeuN^+^ (red) immunofluorescence staining in the hippocampus. Scale bar = 50 µm. **B** Quantification of average NeuN intensity in CA1, CA3 and DG regions. All data are expressed as the mean ± SD. *p < 0.05, *ns* no significance, as indicated. *n* = 5/group. *p* values are based on the one way ANOVA

KA-induced epilepsy is frequently associated with severe neuronal damage in the hippocampus[46]. The morphology and average intensity of NeuN were comparable in WT sham and cKO sham groups. This indicated that the morphology and intensity of neurons in the hippocampus did not change in the physiological conditions after ASK1 cKO. To assess the degree of neuron loss in epileptic mice, we detected the average NeuN intensity in three regions of the hippocampus with immunofluorescence staining (Fig. 8A). Decreased NeuN intensity was observed in the CA1 and CA3 regions after KA administration (Fig. 8B). Comparing with the WT KA group, the cko KA group increased NeuN intensity in the CA1 region (Fig. 8B). Our results indicated that ASK1 deficiency might partially rescue neuronal damage.

### ASK1 conditional knockout blocked the activation of JNK/p38 in Mi/Mϕ

**Fig. 9:**
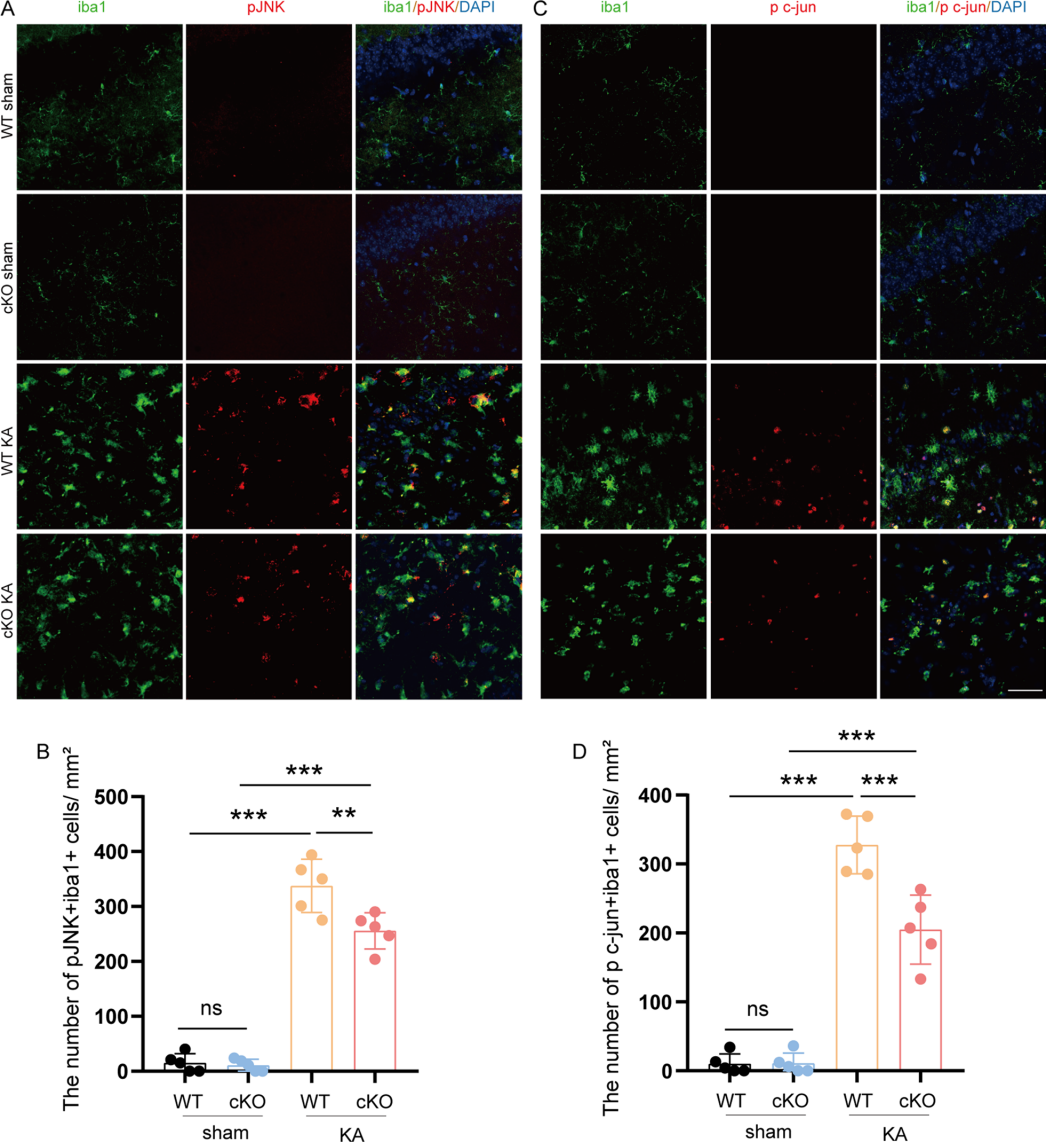
ASK1 cKO decreased the activation of JNK in Mi/Mϕ. **A** Representative images of pJNK^+^/Iba1^+^ (red/green) immunofluorescence staining in the hippocampus. Scale bar = 50 µm. **B** Quantification of the number of pJNK^+^/Iba1^+^ cells. **C** Representative images of p c-jun^+^/Iba1^+^ (red/green) immunofluorescence staining in the hippocampus. Scale bar = 50 µm. **D** Quantification of the number of p c-jun^+^/Iba1^+^ cells. All data are expressed as the mean ± SD. ***p < 0.001, **p < 0.01. *ns* no significance, as indicated, *n* = 5/group*. p* values are based on the one way ANOVA

ASK1 phosphorylates downstream pathways, including c-Jun N-terminal kinase(JNK) and p38. To determine whether ASK1 deletion in Mi/Mϕ affects JNK activation and the transcription factor c-Jun, we performed immunofluorescence staining with antibodies on phosphorylated forms of JNK and c-Jun. Double staining for Iba1 led to lower JNK/c-Jun phosphorylation in the Mi/Mϕ of cKO epileptic mice than in the Mi/Mϕ of WT epileptic mice (Fig. 9A–D). We also conducted double immunofluorescence staining of Iba1 and phosphorylated forms of p38 in the hippocampus. Lower p38 phosphorylation in the Mi/Mϕ of cKO epileptic mice was detected than in the Mi/Mϕ of WT epileptic mice (Additional file 1: Fig. S6). These results suggested that JNK/p38 activation in Mi/Mϕ is dependent on upstream ASK1.

## Discussion

Our study focused on the role of ASK1 in the progression and prognosis of epilepsy. ASK1 is an upstream activator of the mitogen-activated protein kinase (MAPK) signalling pathway that has been linked to neuroinflammation and apoptosis [18]. Current studies have suggested that increased or activated ASK1 can be detected in a variety of neurological diseases [47–49]. Increased ASK1 levels were also found in the hippocampi of epileptic rats by Shinoda et al. [21]; however, the authors failed to detect phosphorylated ASK1 in their study [21]. In our intrahippocampal KA injection model of epilepsy, we found increased levels of phosphorylated ASK1 in the hippocampus during the acute phase of epilepsy. Previous studies have focused on increased ASK1 expression in neurons and the subsequent neuronal apoptosis in epilepsy. Interestingly, our study showed that ASK1 was highly expressed in Mi/Mϕ in the hippocampus, indicating that ASK1 may play a detrimental role in Mi/Mϕ-mediated inflammatory responses.

Microglia are immune cells in the brain [50, 51]. Bone marrow-derived macrophages invade the brain when the blood–brain barrier is disrupted by epilepsy [52]. Most of the innate immune cells in the epileptic brain are microglia and macrophages [52]. These cells survey the surrounding microenvironment and express various immune molecules in response to different diseases[53–55]. When a disease is detected, Mi/Mϕ become reactive and express pro- or anti-inflammatory markers [54, 56–61]. The classically activated Mi/Mϕ produce proinflammatory cytokines (TNFα, IL-1β, IL-6) that lead to histologic damage, while the alternatively activated Mi/Mϕ secrete anti-inflammatory cytokines (IL-13, TGFβ) that promote histological repair [35, 36, 62]. The Mi/Mϕ actively shift between pro- and anti-inflammatory phenotypes and are maintained in a dynamic balance to adapt to the surrounding environment [63–66]. Pro- and anti-inflammatory Mi/Mϕ are observed in epilepsy and determine the outcomes of epilepsy.

Mi/Mϕ-specific ASK1 conditional knockout (ASK1 cKO) mice were used in our study, and we established epileptic models by KA injection. Our study demonstrated that CD206^+^ (anti-inflammatory) Mi/Mϕ was higher and CD16^+^ (proinflammatory) Mi/Mϕ was lower in the hippocampal CA1 region in the ASK1 cKO group than in WT KA group. A similar trend was observed at the mRNA level. The mRNA levels of proinflammatory markers, such as CD16 and TNFα, were lower and the levels of anti-inflammatory markers, such as CCL22 and Arg-1, were higher in the cKO KA group than in the WT KA group. Based on these findings, we concluded that ASK1 deficiency alleviated proinflammatory Mi/Mϕ responses while promoting anti-inflammatory Mi/Mϕ responses in epileptic mice. Our results were consistent with those of Liu L et al. [66]. They found decreased Iba-1^+^iNOS^+^cells (pro-inflammatory) and increased Iba-1^+^Arg-1^+^ cells (anti-inflammatory) via kappa opioid receptor (KOR) activation in epileptic rats. However, Peng J et al. [67] found that the mRNA level of pro-inflammatory cytokines was not reduced in the forebrains and hippocampi of epileptic mice after rosiglitazone treatment. This inconsistency may be due to the use of different epileptic animal models and timepoints.

The high prevalence of neurobehavioral comorbidities in epilepsy is widely acknowledged [4, 7]. These comorbidities severely impact the quality of life and wellbeing of patients, emphasizing the importance of developing effective treatments. However, only limited therapeutic options are currently available [4–7]. In our study, we used a variety of neurobehavioral tests to examine the effect of ASK1 cKO on the neurobehavioral phenotypes. We found that ASK1 deletion in Mi/Mϕ significantly reduced abnormal social and stress related activity and alleviated impaired spatial memory in epileptic mice, but failed to reduce anxiety and depression-like behaviours in epileptic mice. We did not detect increased central time in the open field test. Previous literatures [33, 34] also suggested that the results of animal behavioral experiments were not completely consistent, especially behavioral experiments that detected cognitive functions and emotions. For example, Sun X et al. [33] claimed that the autism-like behaviors of Trio K1431M mutant mice were partially rescued by activation of GABA signaling, with decreased repetitive and stereotyped behaviors in marble burying test and increased time in center zone in open field test after GABA signaling activation. However, the time in open arms was not increased in elevated plus-maze test. Walsh RM et al. [34] performed various behavioral tests, they also found that Phf8 KO mice failed to show improvements in all tests. For instance, they did not detect decreased anxiety and depression in light–dark box assay and tail suspension test after Phf8 KO. The results of these tests were influenced by various factors, including age and sex of the experimental animals, light exposure in the experimental environment and so on. Our results showed that ASK1 cKO significantly reduced abnormal social and stress related activity and alleviated impaired spatial memory in epileptic mice, but failed to reduce anxiety and depression-like behaviors in epileptic mice.

However, a few questions warrant further study. The seizures recorded by EEG traces were quite important for epilepsy research. Though many literatures [68–70] have reported that behavioral seizures were often accompanied by electrographic seizures in EEG, the results of behavioral seizures and electrographic seizures were always consistent, we only do the assessment of behavioral seizures in our study, it is the limitation. EEG traces to detect electrographic seizures are required in our future study to verify the effect of ASK1 cKO. Second, it was difficult to determine the specific role of ASK1 in microglia and macrophages because ASK1 was genetically depleted in both cell lines. However, microglial-specific depletion of ASK1 could be achieved after 4 weeks of rest following tamoxifen administration [71]. During these 4 weeks, ASK1 was restored in macrophages because of the rapid turnover of blood monocytes, while ASK1 was not updated in microglia in a timely manner [52]. Thorough studies on the specific role of microglial ASK1 should be performed. In addition, ASK1 signalling pathways warrant further investigation in future research. Moreover, we only used two animal models: for instance, intrahippocampal KA injection. However, differences may exist among different epileptic animal models [64]. To confirm the neuroprotective role of ASK1 deletion, ASK1 conditional knockout in epileptic mice by other means such as intraperitoneal injections of pilocarpine, should be studied. Additionally, we did not explore whether ASK cKO mice show abnormal neuronal activity in baseline condition by electrophysiological sEPSCs test, which is one of limitations in our study, though the morphology and intensity of neurons in the hippocampus did not change in the physiological conditions after ASK1 cKO, and previous literatures [72, 73] have reported that ASK1 deficiency did not influence the normal neuronal activity in physiological condition.

## Conclusion

In conclusion, this study emphasized the importance of ASK1 in modulating the inflammatory responses of microglia/macrophages in epilepsy. ASK1 conditional deletion reduced neuroinflammation and neuron loss while ameliorating seizure frequency, as well as social and cognitive comorbidities. These results suggested that ASK1 in microglia/macrophages could be a promising therapeutic agent for inflammatory responses in epilepsy.

## Supplementary Information

**Additional file 1: Figure S1.** The expression of ASK1 in microglia/macrophages in cortex and striatum of epileptic mice. **Figure S2.** ASK1 cKO reduced ASK1 expression in microglia/macrophages. **Figure S3.** ASK1 cKO significantly increased the latency of PTZ induced seizures. **Figure S4.** ASK1 cKO failed to reduce anxiety and depression-like behaviors in epileptic mice. **Figure S5. **ASK1 cKO decreased the total length of processes in Mi/Mϕ. **Figure S6.** ASK1 cKO decreased the activation of p38 in Mi/Mϕ.

## Data Availability

The datasets used and/or analyzed during the current study are available from the corresponding author on reasonable request.

## Abbreviations

ASK1: Apoptosis signal-regulating kinase1
JNK: C-Jun N-terminal kinase
IL-1β: Interleukin 1β
IL-6: Interleukin 6
TNF-α: Tumor necrosis factor α
Arg1: Arginase1
CCL22: C–C motif chemokine ligand 22
IL13: Interleukin 13
TGFβ: Transforming growth factor β
DG: Dentate gyrus
NeuN: Neuronal nuclei
SRS: Spontaneous recurrent seizures
SE: Status epilepticus
KA: Kainic acid
WT: Wild type
cKO: Conditional knockout
Mi/MΦ: Microglia/macrophages
AP: Anteroposterior
ML: Mediolateral
DV: Dorsoventral
