# Supplementary material for: Conditional knockout of ASK1 in microglia/macrophages attenuates epileptic seizures and long-term neurobehavioural comorbidities by modulating the inflammatory responses of microglia/macrophages
Source: J Neuroinflammation. 2022 Aug 8;19:202. doi: 10.1186/s12974-022-02560-5 (PMC9361603; doi:10.1186/s12974-022-02560-5)
Supplement: Supplementary file 1 — Additional file 1: Figure S1. The expression of ASK1 in microglia/macrophages in cortex and striatum of epileptic mice. Figure S2. ASK1 cKO reduced ASK1 expression in microglia/macrophages. Figure S3. ASK1 cKO significantly increased the latency of PTZ induced seizures. Figure S4. ASK1 cKO failed to reduce anxiety and depression-like behaviors in epileptic mice. Figure S5. ASK1 cKO decreased the total length of processes in Mi/Mϕ. Figure S6. ASK1 cKO decreased the activation of p38 in Mi/Mϕ. [file 12974_2022_2560_MOESM1_ESM.docx]

Supplementary Figures


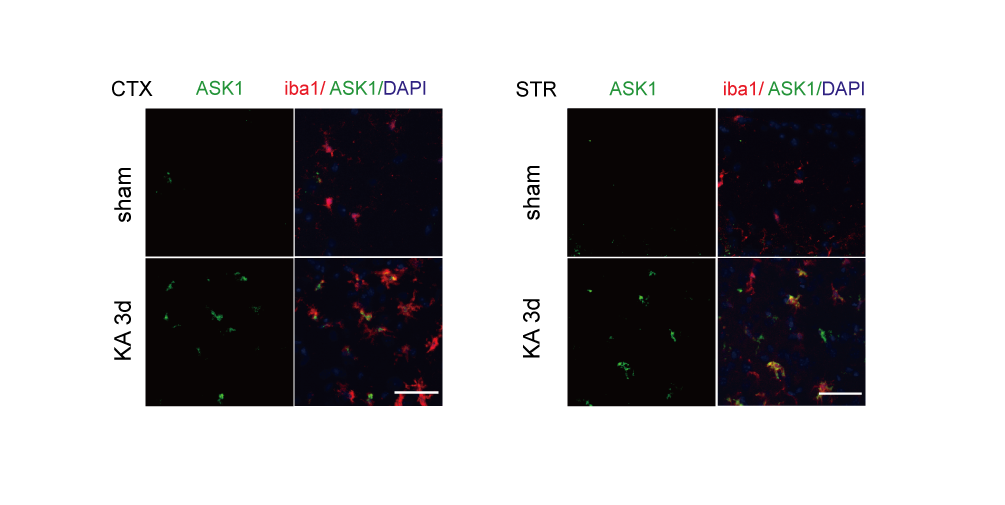


**Figure S1. The expression of ASK1 in microglia/macrophages in cortex and striatum of epileptic mice.** Representative images of ASK1(green), Iba1(red) , and DAPI (blue) in cortex and striatum 3 day post seizures, Scale bar = 50 µm.


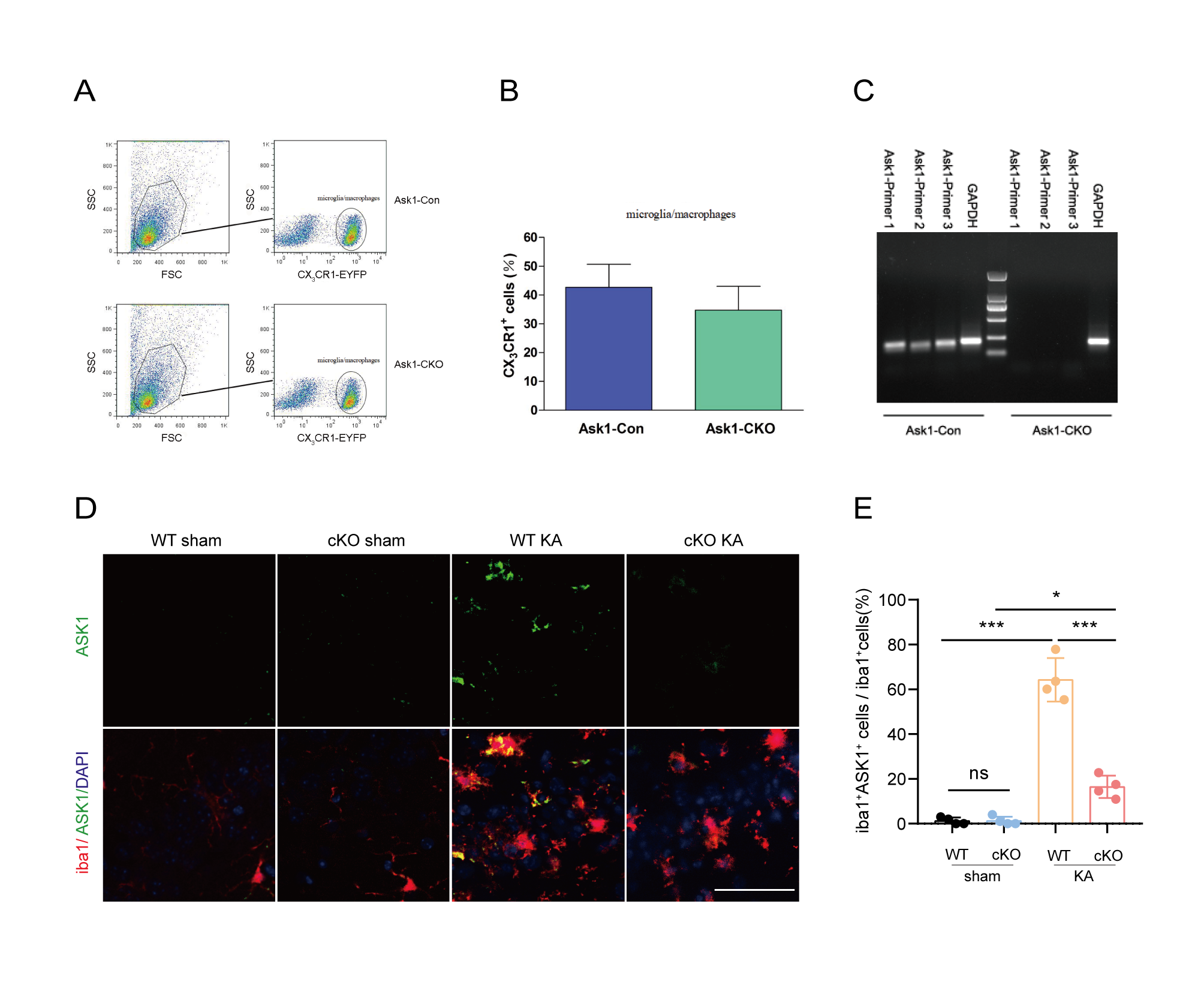


**Figure S2. ASK1 cKO reduced ASK1 expression in microglia/macrophages.** (A). Gating strategy for sorting Microglia/macrophages (CX3CR1^+^) by flow cytometry. (B). Quantification of Microglia/macrophages in the brain of WT sham or cKO sham groups. (C). Agarose gel electrophoresis detected the ASK1 PCR product. (D). Representative images of ASK1(green) and Iba1(red) immunofluorescence staining 3 day post seizures, Scale bar = 55 µm, n = 4/group. (E). Quantification of the number of ASK1^+^/Iba1^+^ cells. All data are expressed as the mean ± SD. ****p* < 0.001, and **p* < 0.05, ns: no significance, as indicated. *p* values are based on the one way ANOVA.


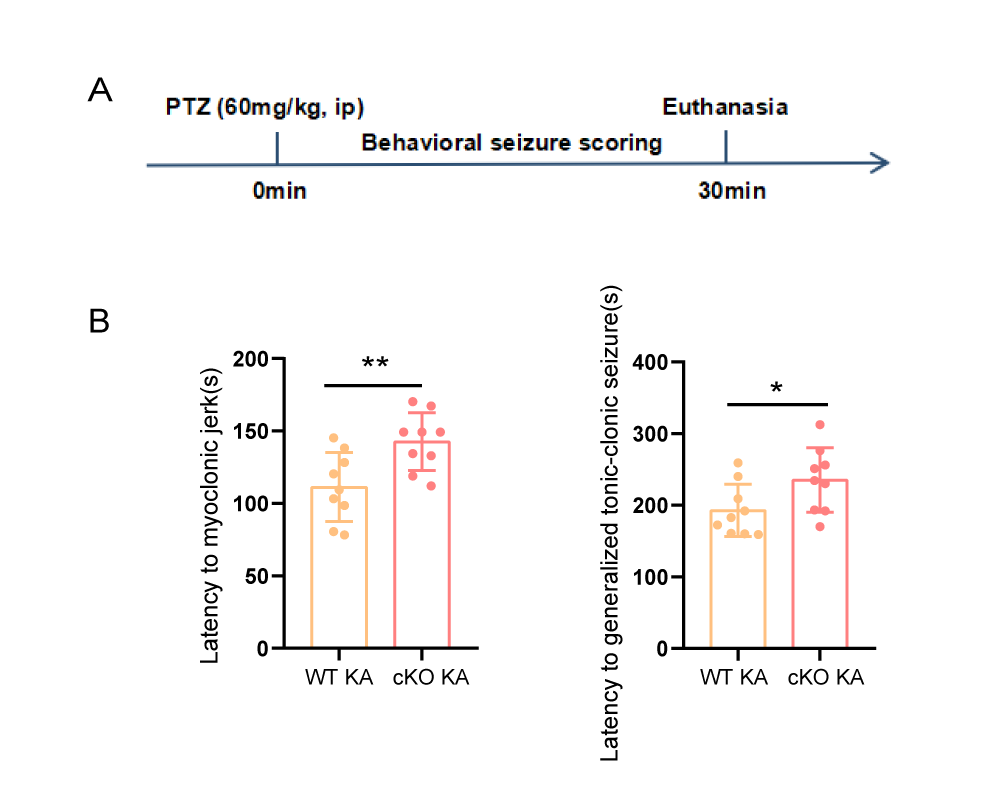


**Figure S3. ASK1 cKO significantly increased the latency of PTZ induced seizures.** (A). The experimental design was displayed in timeline. (B). The latency to the onset of first myoclonic jerk and first generalized tonic-clonic seizure (GTCS) were recorded after PTZ administration. n=9/group. All data are expressed as the mean ± SD. ***p* < 0.01, and **p* < 0.05, as indicated. *p* values are based on the Student’s t test.


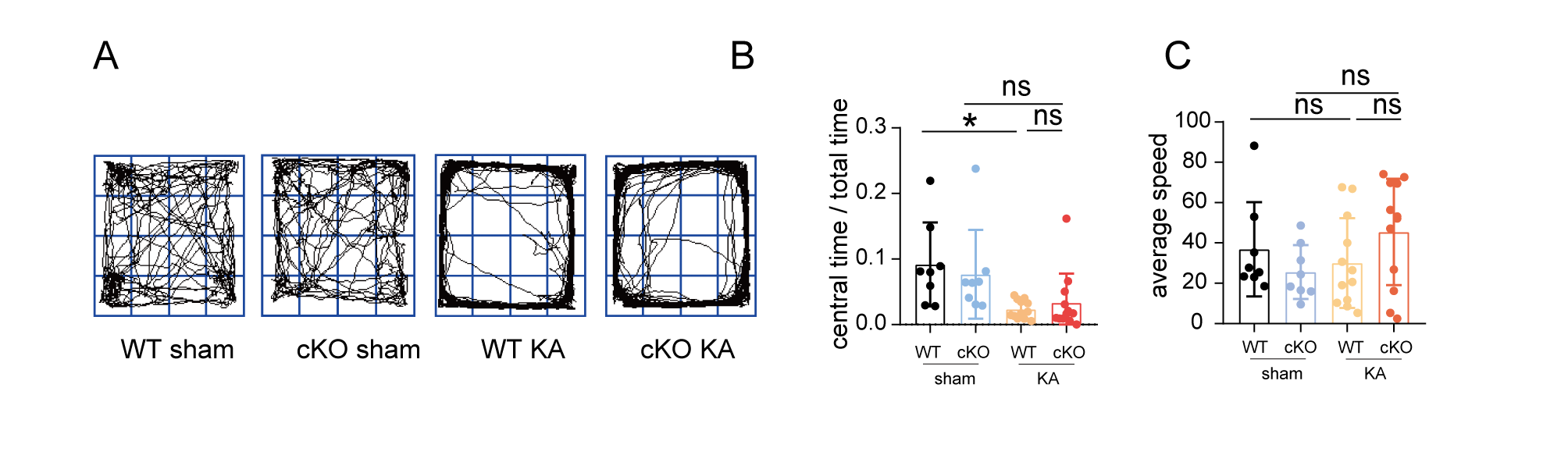


**Figure S4. ASK1 cKO failed to reduce anxiety and depression-like behaviors in epileptic mice.** (A). Representative track chart of 4 groups in the open field test. (B). Quantification of the ratio of time spent in the central area. (C). Quantification of the average speed. All data are expressed as the mean ± SD and analyzed using the Kruskal-Wallis ANOVA. * *p* < 0.05, ns: no significance, as indicated. n = 8 in the WT sham or cKO sham groups, n = 12 in the WT KA or cKO KA groups.


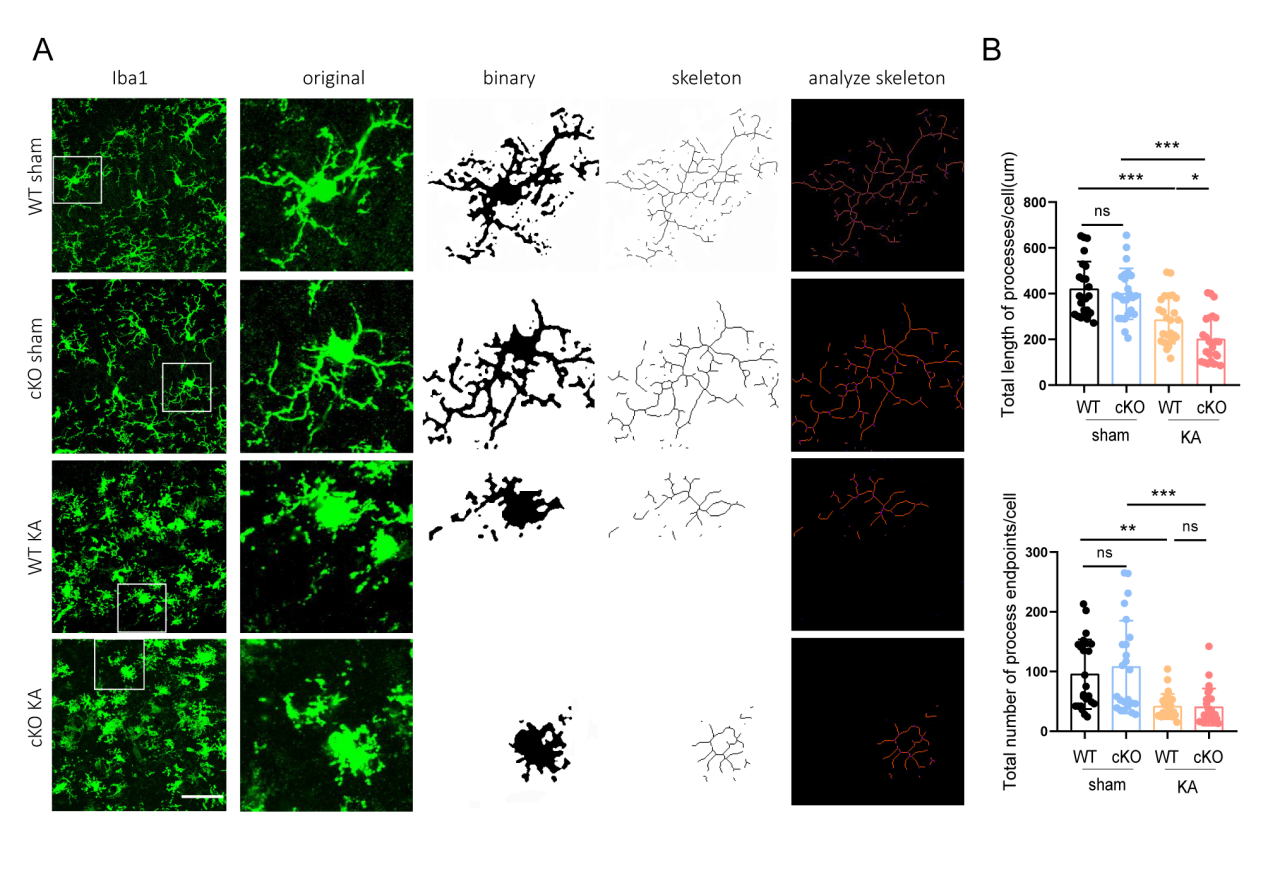


**Figure S5. ASK1 cKO decreased the total length of processes in Mi/Mφ.** (A). Representative images of Iba1 immunofluorescence staining. Scale bar = 50 µm. (B). The skeleton reconstruction by image Fiji, the protrusion length and the number of protrusions of each cell were analyzed. All data are expressed as the mean ± SD. ****p* < 0.001, ** *p* < 0.01, and **p* < 0.05, ns: no significance. n = 24 /group.


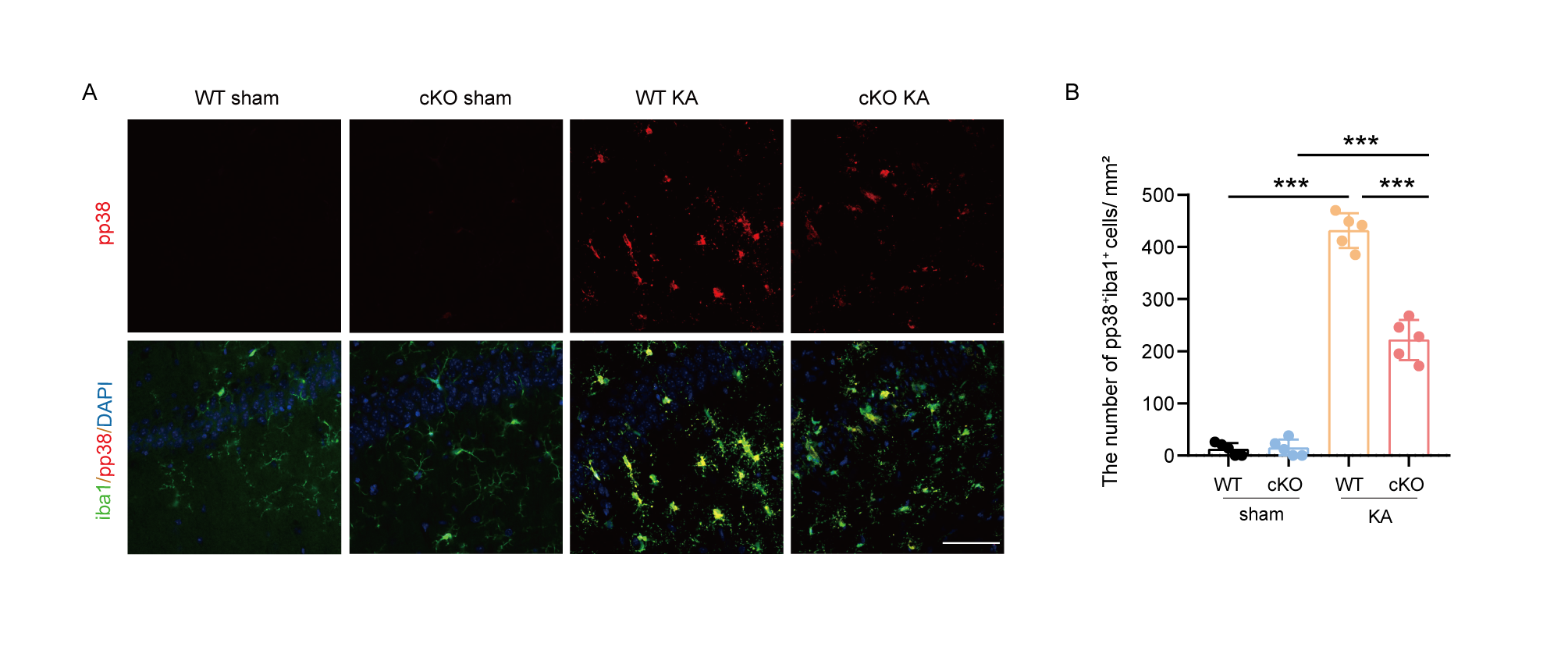


**Figure S6. ASK1 cKO decreased the activation of p38 in Mi/Mφ.** (A). Representative images of pp38^+^/Iba1^+^ (red/green) immunofluorescence staining in the hippocampus. Scale bar = 55 µm. (B). Quantification of the number of pp38^+^/Iba1^+^ cells. All data are expressed as the mean ± SD. ****p<* 0.001, as indicated. n = 5/group. *p* values are based on the one way ANOVA.
